# Absence of *Glutathione S-Transferase Theta 1* Gene Is Significantly Associated With Breast Cancer Susceptibility in Pakistani Population and Poor Overall Survival in Breast Cancer Patients: A Case-Control and Case Series Analysis

**DOI:** 10.3389/fonc.2021.678705

**Published:** 2021-12-06

**Authors:** Sadia Ajaz, Sani-e-Zehra Zaidi, Saleema Mehboob Ali, Aisha Siddiqa, Muhammad Ali Memon, Sadaf Firasat, Aiysha Abid, Shagufta Khaliq

**Affiliations:** ^1^ Dr Panjwani Center for Molecular Medicine and Drug Research (PCMD), International Center for Chemical and Biological Sciences (ICCBS), University of Karachi, Karachi, Pakistan; ^2^ Department of Human Genetics and Molecular Biology, University of Health Sciences, Lahore, Pakistan; ^3^ Atomic Energy Medical Centre (AEMC), Jinnah Postgraduate Medical Centre (JPMC), Karachi, Pakistan; ^4^ Centre for Human Genetics and Molecular Medicine, Sindh Institute of Urology and Transplantation (SIUT), Karachi, Pakistan

**Keywords:** breast cancer, molecular epidemiology, polymorphism, null genotype, *GSTT1-*absent, *GSTT1-*present

## Abstract

**Purpose:**

Deletion of Glutathione S-Transferase Theta 1 (GSTT1) encoding gene is implicated in breast cancer susceptibility, clinical outcomes, and survival. Contradictory results have been reported in different studies. The present investigation based on a representative Pakistani population evaluated the *GSTT1*-absent genotype in breast cancer risk and prognosis.

**Methods:**

A prospective study comprising case-control analysis and case series analysis components was designed. Peripheral blood samples were collected from enrolled participants. After DNA extraction, *GSTT1 g*enotyping was carried out by a multiplex PCR with *β-globin* as an amplification control. Association evaluation of *GSTT1* genotypes with breast cancer risk, specific tumor characteristics, and survival were the primary endpoints.

**Results:**

A total of 264 participants were enrolled in the molecular investigation (3 institutions). The study included 121 primary breast cancer patients as cases and 143 age-matched female subjects, with no history of any cancer, as controls. A significant genetic association between *GSTT1*-absent genotype and breast cancer susceptibility (*p*-value: 0.03; OR: 2.13; 95% CI: 1.08-4.29) was reported. The case-series analysis showed lack of association of *GSTT1* genotypes with menopause (*p-*value: 0.86), tumor stage (*p*-value: 0.12), grade (*p*-value: 0.32), and size (*p*-value: 0.07). The survival analysis revealed that *GSTT1*-absent genotype cases had a statistically significant shorter overall survival (OS) than those with the *GSTT1*-present genotype cases (mean OS: 23 months *vs* 33 months). The HR (95% CI) for OS in patients carrying *GSTT1*-absent genotype was 8.13 (2.91-22.96) when compared with the *GSTT1*-present genotype.

**Conclusions:**

The present study is the first report of an independent significant genetic association between *GSTT1*-absent genotype and breast cancer susceptibility in a Pakistani population. It is also the foremost report of the association of this genotype with OS in breast cancer cases. Upon further validation, *GSTT1* variation may serve as a marker for devising better population-specific strategies. The information may have translational implications in the screening and treatment of breast cancers.

## Introduction

Glutathione is present in all living cells. Physiologically, it performs three important functions: protection of thiol groups in proteins from oxidation, intracellular redox buffering, storage for sulphur-containing cysteine. These functions are dependent upon the catalysis by Glutathione S-Transferases (GSTs), E.C. 2.5.1.18. Consequently, GSTs play a major role in the detoxification of potent endogenous and exogenous carcinogens (1). These enzymes constitute a superfamily of isoenzymes including GST- theta 1. *GSTT1* gene is located on chromosome 22q11.2. It encodes the enzyme, which is involved in the conjugation of reduced glutathione to certain electrophiles and hydrophobic compounds (2). Ultimately, such toxic substrates may be removed from the body.

The absence of the *GSTT1* gene, also known as homozygous deletion or null genotype and herein referred to as *GSTT1*-absent, has been reported with varying frequencies in different populations (3). The carriers of the *GSTT1*-absent genotype are unable to metabolize some mutagenic carcinogens (4). The deletion has been correlated with ovarian, bladder, colon, oral, lung, and pediatric cancers among different populations (5–10). It is a candidate genetic marker for cancer risk, prognosis, and treatment response. In the case of breast cancers, the independent contribution of *GSTT1* null genotype to susceptibility, tumor characteristics, and response to prescribed regimens remains inconclusive in different populations across the world (11–13).

In Pakistan, the age-standardized rate (ASR) of the female breast cancer incidence is among the highest in Asia (34.4 per 100,000), whereas the mortality rate is one of the highest in the world (18.8 per 100,000) (14, 15). Therefore, it is essential to identify the underlying factors in breast cancer etiology and prognosis.

Two previous studies from Pakistan (16, 17) report no independent association between the absence of the *GSTT1* gene and breast cancer susceptibility. Both the studies were published from the Punjab area. However, Pakistan shows ethnicity-specific genetic variation across its region (18). Furthermore, the small sample size, and conflicting frequencies in controls: 18.7% (16) *vs* 31.4% (erroneously reported as 16% in one of the studies) (17), limit the applicability of these conclusions.

This prospective observational molecular study was designed based on the biological plausibility of *GSTT1* deletion in carcinogenesis. It addresses the paucity and contradiction in the available data from a region that has frequent and aggressive breast tumors. The first component of the study, the case-control analysis, evaluated the contribution of the *GSTT1* gene in breast cancer risk. Simultaneously, the second part, comprising case series analysis, investigated the contribution of *GSTT1* genotypes to specific tumor characteristics and breast cancer survival after standard treatment.

## Materials and Methods

### Study Design and Participant Enrollment

**Figure 1:**
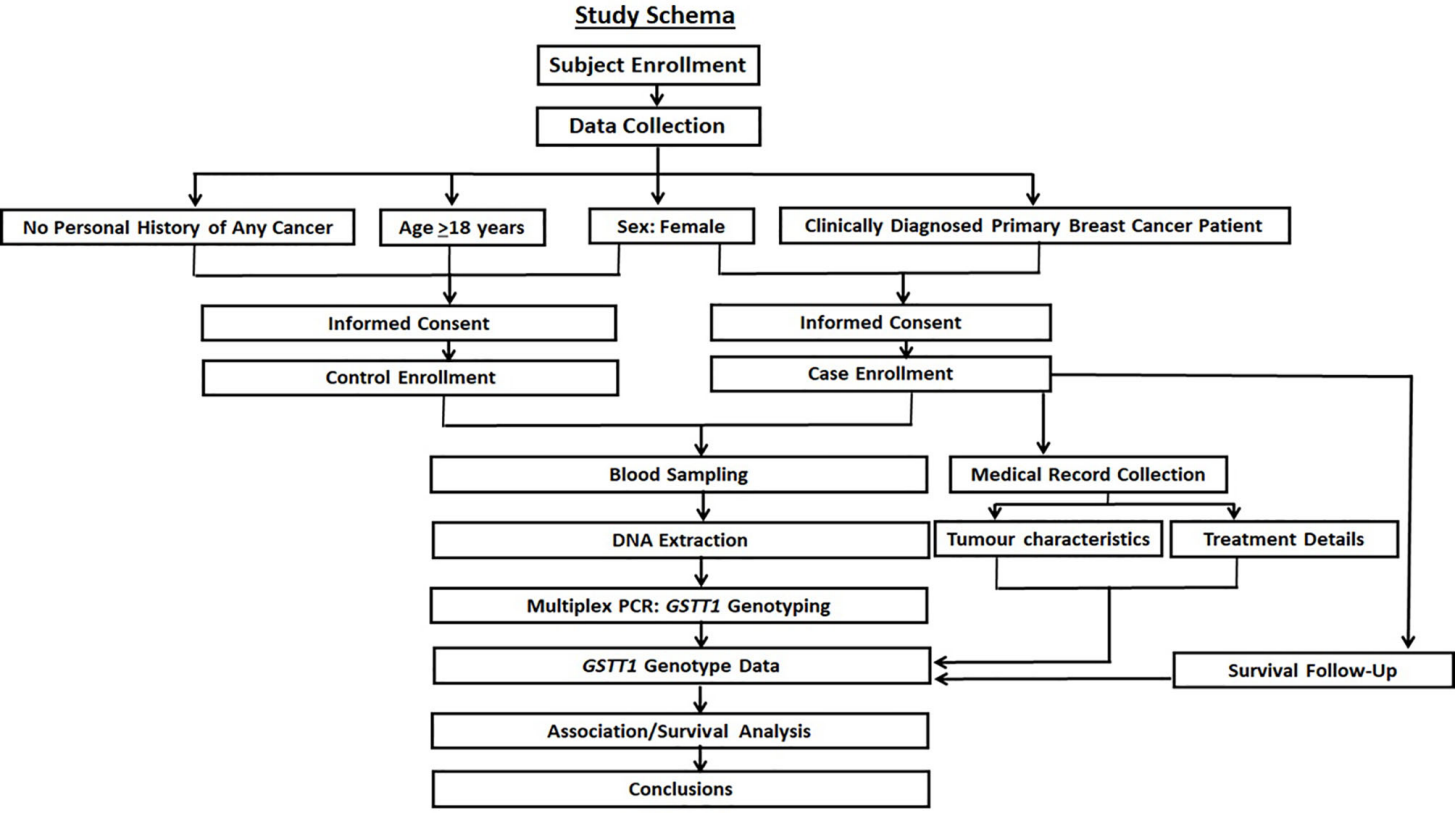
The study schema. The prospective recruitment of cases with age-matched female controls is shown along with the collection of specified molecular, clinical, and survival data. The objective was to investigate the contribution of *GSTT1* variation in breast cancer risk, tumor characteristics, and survival after standard treatment.

The overall study schema is shown in **Figure 1**.

The patients were recruited from the Atomic Energy Medical Centre (AEMC), Jinnah Postgraduate Medical Centre (JPMC), Karachi, Pakistan. The cases were clinically diagnosed primary breast cancer patients. The treatment included radiotherapy at the aforementioned participating institution following chemotherapy and surgery. The latter two components of treatment were carried out at hospitals other than AEMC. The details of control enrolment have been published elsewhere (19), with the modification that only age-matched (≥ 18 years), female participants’ data were included in the present study. All the subjects were recruited in Karachi, Pakistan and therefore, the distribution of ethnicities was the same in cases and controls. Sindhi, a self-defined Urdu-speaking ethnicity, Pathan, and Punjabi were the main ethnic groups. The research involved human participants and followed the provisions of the Declaration of Helsinki and its amendments. Research protocols were approved by the independent Ethics Review Committees of all the relevant institutions. The present study follows the reporting recommendations for tumor marker prognostic studies (REMARK) (20, 21) (**Supplementary Table 1**).

### Data Collection

Information regarding age was recorded for all the participants. Patients’ family history, age at menarche, menopause (if applicable), and obstetrics and gynecology history were recorded in a questionnaire. In cases, tumor node metastasis (TNM) staging and histological grading were carried out according to the Union Internationale Contre le Cancer (UICC) recommendations (22). Data on tumor characteristics (tumor stage, grade, and size) and histology were obtained from the patients’ hospital medical files. Information relating to the parameters that were analyzed was documented in the majority of the hospital records. Three-year survival data were collected through telephonic follow-up. The missing information was due to: (i) the return of patients to their towns/villages after treatment at Karachi; (ii) erroneous contact information; and (iii) no response.

### Sample Collection and DNA Extraction

All the participants volunteered 8-10ml of venous blood sample, which was collected in ACD-coated vacutainers (BD Vacutainer^®^ BD Franklin Lakes NJ USA). Samples from the cases were collected at the time of radiotherapy, post-mastectomy, and chemotherapy treatment. The blood samples were either processed immediately or stored at 4°C until DNA extraction.

**Figure 2:**
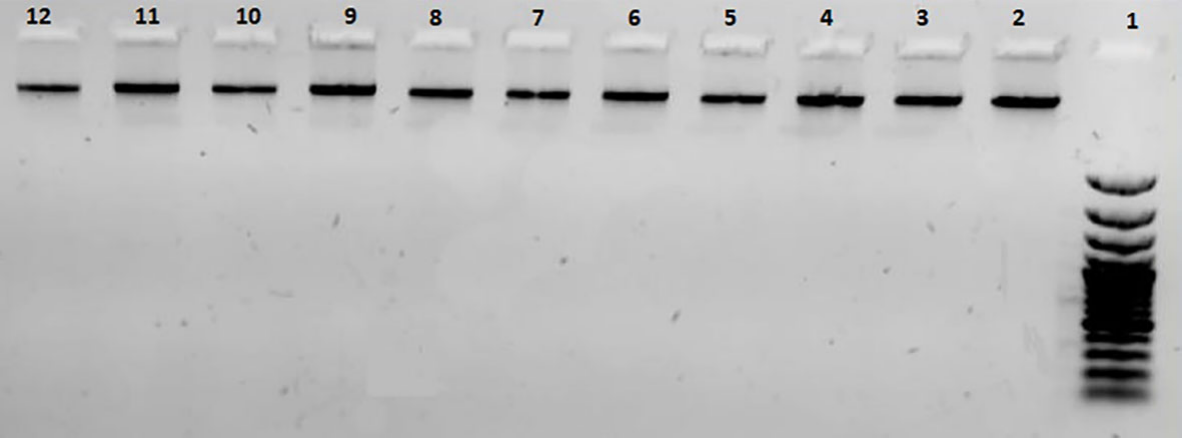
A representative 0.7% agarose gel used for quality control of extracted DNA samples. Lane 1: 100bp DNA ladder; lanes 2-12: DNA samples.

DNA was extracted from the white blood cells according to the standard phenol-chloroform method (23). It was quantified spectrophotometrically (Beckman Coulter™ DU^®^ 530). The quality control cut-off for the 260/280 ratio was between 1.7-1.99. DNA quality was also analyzed by 0.7% agarose gel electrophoresis followed by UV visualization using a gel imaging system (Azure c300^®^ biosystems). No fragmentation or smearing was observed in any of the samples (**Figure 2**).

The working dilutions for experiments were prepared at room temperature and stored at 4°C. The stock DNA samples were stored at -20°C.

### Genotyping

#### Cases

*GSTT1* genotyping was carried out by a multiplex polymerase chain reaction (PCR) with *β-globin* as an amplification control. The primer sequences have been published earlier (17).

**Figure 3:**
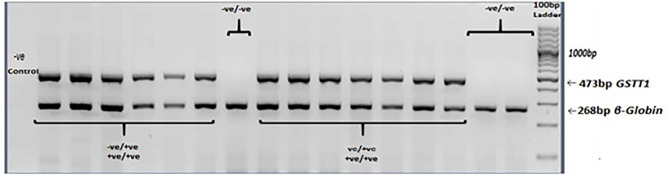
*GSTT1* genotyping. Agarose gel electrophoresis (2%) of multiplex PCR amplified products: *GSTT1*-absent (–ve/-ve) genotype did not show amplification of a 473bp fragment. *β-globin* gene was included as an amplification control.

PCR was carried out with a *Taq* DNA polymerase kit (Thermofisher Scientific Inc.). Total PCR reaction mix (10µl) consisted of 1X PCR buffer, 0.9mM MgCl_2_, 0.5mM dNTPs, 1.5U/µl *Taq* polymerase, 1.8µM primers each for *GSTT1* and *β-globin* genes, and 70ng DNA. The PCR conditions were: initial denaturation at 94°C for 5 minutes, followed by 40 cycles of 94°C for 45 seconds, annealing at 60°C for 45 seconds, and extension at 72°C for 45 seconds. The final extension was carried at 72°C for 5 minutes. Amplicons were analyzed under UV on 2% agarose gel, which was stained with ethidium bromide. A fragment of 473bp indicated the *GSTT1*-present genotype. The *GSTT1*-absent genotype did not show this amplification. The amplification of the β-globin gene served as the control for successful PCR. A negative control was included in all the genotyping experiments (**Figure 3**).

The results of genotyping were confirmed by double-blind evaluation, the inclusion of replicates, and negative controls.

#### Controls

The genotyping in controls has been described elsewhere (19). It identifies homozygous *GSTT1-*present, heterozygous *GSTT1-*present/absent, and homozygous *GSTT1-*absent genotypes. In the final observation, the *GSTT1*-present allele was determined by an amplicon of 466bp, while the *GSTT1*-absent allele was identified by an amplicon of 1,460bp.

### Treatment of Breast Cancer Patients

All the participating cases underwent a mastectomy, adjuvant chemotherapy, and/or radiotherapy. Before the start of chemotherapy, echocardiography was done to assess cardiac function (ejection fraction cut-off for the start of doxorubicin based chemo was >55%). A test dose of docitaxel was given to rule out hypersensitivity before the first cycle. Neurological assessment was undertaken during taxane (paclitaxel) cycles. Chemotherapy-related toxicities were assessed after every cycle according to Common Toxicity Criteria of the National Cancer Institute (NCI-CTC, version 2.0) (24). “Severe toxicity” was defined as hematological or gastrointestinal toxicity of grades 3–4.

The chemotherapy regimen included: Adriamycin-Cyclophosphamide x4 followed by taxane x4 (docitaxel or paclitaxel): Doxorubicin 60mg/m^2^ on day 1, cyclophosphamide 600mg on day 1, paclitaxel 175mg/m^2^ on day 1 OR docitaxel 100mg/m^2^ on day 1, repeated every 3 weeks.

Complete blood count, liver function test, and renal function test were carried out to assess the treatment response.

### Statistical Analysis

The allele distribution for *GSTT1* polymorphism in the controls was assessed for Hardy-Weinberg equilibrium (25). The statistical tests for association analysis were carried out by using Statistical Package for Social Science (SPSS) for Windows v.19.0 (SPSS, Inc., Chicago, Illinois, USA) and online OpenEpi software (26).

In the case-control investigations, data for the *GSTT1* genotype was obtained for all the participants, except two cases, where no amplification was recorded. The age-matching between cases and controls was analyzed by Student’s t-test for independent samples with the assumption of unequal variances. To achieve 80% power at a two-sided level of significance, various odds ratios (OR) of genetic risk due to *GSTT1* polymorphism for breast cancers were calculated. The accrual of 260 participants (matched cases and controls) allows for the identification of OR≥2 for *GSTT1* variation with the *GSTT1*-absent frequency of 0.24 [the median value of the reported prevalence in controls from Pakistan (16–18, 27–38) was used for the calculations (39)]:


$$\text{n}=\frac{(z_{a}\sqrt{2\bar{p}\bar{q}})+z_{\beta }\sqrt{p_{1}q_{1}+p_{0}q_{0}}{)}^{2}}{{\left(p_{1}-p_{0}\right)}^{2}}$$

Where,


$$\begin{array}{c} \bar{p}=\frac{p_{1}+p_{0}}{2}\\ \bar{q}=1-\bar{p}\\ q_{1}=1-p_{1}\\ q_{0}=1-p_{0} \end{array}$$

n = number of subjects in each group

z_α_ = Corresponding to α [level of significance (95%)] = 1.96

z_β_= Corresponding to β (Probability of type II error. Power of study is 80%, 1- β = 0.2)

p_0_ = proportion of exposure among control groups (*prevalence of the polymorphism in general population without breast cancer).

p_1_ = proportion of exposure among cases based on the formula including odds ratios associated with exposure.

The missing information for case series analysis is itemized in the relevant tables in the results section.

The primary objective was the investigation of *GSTT1* polymorphism association/s with breast cancer susceptibility, the selected clinical parameters, and survival. The data were assessed by Pearson χ^2^ test. The ORs were tabulated with a 95% confidence interval (95% CI) to evaluate the strength of the associations. *Post hoc* power analysis was carried out to assess the strength of the study (40, 41).

The overall survival (OS) and hazard ratios (HR) with 95% CI were assessed by the Kaplan-Meier method using MedCalc software v.19.2.6 (42, 43). In all the statistical tests, p-values <0.05 were considered to be significant.

## Results

### Participants’ Information and Clinical Data of Patients

The total number of patients diagnosed with primary breast cancer disease was 121, whereas the total number of age- and gender-matched controls was 143. Characteristics of the 264 participants included in the study are presented in **Table 1**.

**Table 1:** Participant information and clinico-pathological data of breast cancer patients.

Sr. No.	Characteristic	Value	*p-value*
1.	No. of participants (cases/controls)	264 (121/143)	N/A
2.	Mean Age [cases/controls: years ± standard error of mean	44.48 ± 0.95/45.62+0.58	0.306
3.	Mean Age at menopause: years (cases)	42.03 ± 0.94	0.992
4.	Tumor Stage (n*= 84)		
	I and II	I: 3 (4%) and II: 30 (36%)	0.024**
	III and IV	III: 46 (55%) and IV: 5 (5%)	
4.	Tumor Size (n*=102)		
	<2cm	14 (14)	
	2-5cm	58 (57)	<0.01**
	>5cm	30 (29)	>0.05
5.	Tumor grade (n*=107)		
	G1 and G2	G1: 1 (1%) and G2: 46 (43)	>0.05
	G3 and G4	G3: 58 (54%) and G4: 2 (2%)	
6.	Treatment Response (n*=97)		
	Positive	64 (66%)	<0.01**
	Negative (relapse and/or death)	33 (34%)	
7.	3-Year Survival (n*=97)		
	Alive	71 (73%)	<0.01**
	Expired	26 (27%)	

*available data from 121 patients (missing data has been explained in the methodology section); **statistically significant.

The mean age of the patients was 44.48 ± 0.95 years, whereas, for the controls, the mean age was 45.62 ± 0.58 years. All patients presented with invasive ductal carcinoma (IDC) of the breast. Majority of the patients had advanced tumor stage (stages III and IV; *p-*value: 0.024**), tumor size of >2cm (*p*-value: <0.01**), and high tumor grade (grades 3 and 4; *p*-value: >0.05).

### Association Between *GSTT1* Polymorphism and Breast Cancer Risk

The allelic and genotypic frequencies of *GSTT1* polymorphism in controls are shown in **Table 2**. The proportions were in Hardy-Weinberg equilibrium.

**Table 2:** Distribution of *GSTT1* genotypes and allele frequencies (with standard errors) in age- and gender-matched controls. Assessment of HWE test in controls.

*GSTT1* Polymorphism	Controls (n = 143)
*Genotypes*	
*GSTT1*-present/*GSTT1*-present	66 (46%)
*GSTT1*-present/*GSTT1*-absent	61 (43%)
*GSTT1*-absent/*GSTT1*-absent	16 (11%)
*Allele Frequencies*	
p[*GSTT1*-present]	0.67 ± 0.042
q[*GSTT1*-absent]	0.33 ± 0.042
*Hardy-Weinberg Equilibrium* (*HWE*) *Test*	
χ^2^	0.11
*P*-value	NS (0.74)

Associations between the *GSTT1* genotypes and breast cancer susceptibility are presented in **Table 3**.

**Table 3:** Distribution of *GSTT1* genotypes in controls, breast cancer patients, and the association analysis with breast cancer risk*.

*GSTT1* Polymorphism	Controls (n = 143)	Breast Cancer Patients (n = 118^¶^)	χ^2^ Test (*p-*value)	OR (95% CI)
*GSTT1*-absent/*GSTT1*-absent	16 (11%)	25 (21%)	χ^2^ = 4.88; p=0.03**	2.13 (1.08-4.29)**
*GSTT1*-present/*GSTT1*-present & *GSTT1*-present/*GSTT1*-absent	127 (89%)	93 (79%)		

^*^ Post-hoc power of the study: 60.1% **^¶^** Genotype could not be determined for three samples; **statistically significant.

The comparison of *GSTT1*-present genotype with *GSTT1*-absent genotype in cases and controls revealed that *GSTT1*-absent genotype was significantly associated with risk for breast cancers (*p*-value: 0.03). The OR were 2.13 (95% CI: 1.08 - 4.29).

### Lack of Association Between *GSTT1* Polymorphism and Specific Parameters

In the case series analysis, the present study did not report any statistically significant association between the absence of the *GSTT1* gene and the studied tumor characteristics, i.e., stage, grade, and size. The *p-*values were 0.12, 0.32, and 0.07, respectively. Similarly, the study did not find any correlation between the *GSTT1* genotype and age at menopause (p-value: 0.86).

### Association of the *GSTT1* Polymorphism and OS in Breast Cancer Patients

**Figure 4:**
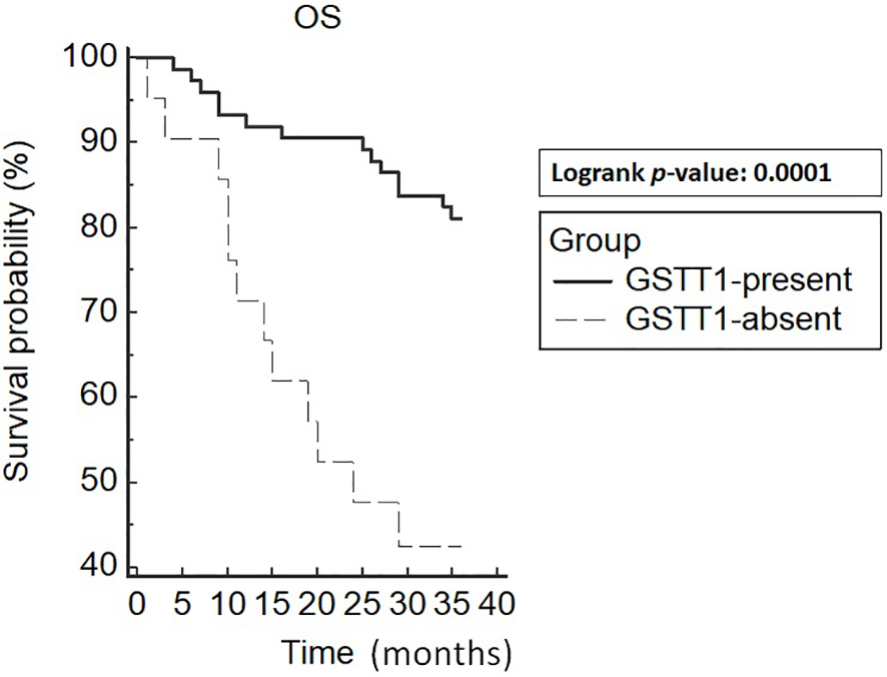
Kaplan-Meier curve demonstrating the overall survival (OS) based on genotypes of *GSTT1*. The mean OS was 33 months (95% CI: 30.96-34.65) in GSTT1-present genotype carriers and 23 months (95% CI: 17.90-28.59) in *GSTT1*-absent genotype carriers; *p*-value: 0.0001.

As shown in **Figure 4**, *GSTT1*-present carriers had 10 months’ longer survival (mean OS: 33 months; 95% CI: 30.96-34.65) than those with *GSTT1*-absent genotype (mean OS: 23 months; 95% CI 17.90-28.59); *p*-value: 0.0001. The HR with 95% CI for OS in patients carrying *GSTT1*-absent genotype was 8.13 (2.91-22.96) with *GSTT1*-present genotype as the reference variable (**Table 4**).

**Table 4:** Associations between *GSTT1* genotypes and overall survival (OS).

*GSTT1* Polymorphism	No. (95)	OS			
		Mean (months)	*p*-value	HR	95% CI
*GSTT1*-absent/*GSTT1*-absent	21 (22%)	23	0.0001**	8.13	2.91-22.96**
*GSTT1*-present/*GSTT1*-present & *GSTT1*-present/*GSTT1*-absent	74 (78%)	33		Ref.	

**statistically significant.

## Discussion

In the present study, the association/s of *GSTT1* genotypes with breast cancer-related parameters were evaluated. In this study, we report a significant association of *GSTT1*-absent genotype with increased breast cancer risk in a representative sample from a Pakistani population. The OR were 2.13 (95% CI: 1.08 – 4.29). We also report a significant difference in the survival duration between *GSTT1*-present and *GSTT1-*absent carriers: mean OS*_GSTT1_*_-present_: 33 months (95% CI: 30.96-34.65) *vs* mean OS*_GSTT1_*_-absent_: 23 months (95% CI: 17.90-28.59). The present analysis is the first report of population-specific associations between *GSTT1* genotypes and the specific factors associated with breast cancers.

The incidence of breast cancers varies across the globe. The highest estimated age-standardized incidence rates have been reported in Belgium (113.2 per 100,000), while the lowest was reported in Bhutan (5.0 per 100.000). Furthermore, the highest estimated age-standardized mortality rates are reported from Barbados (42.2 per 100,000), while the lowest is reported from Bhutan (2.6 per 100,000) (15). The known risk factors such as age, family history, different reproductive parameters, and obesity account for only one-third of the risk for breast cancers (11, 44). In addition, the reason(s) for high mortality rates across specific populations need to be determined (45–47).

A number of genes are likely involved in breast cancer characteristics, with the possibility of gene-environment interactions (48). The quantitative contributions of such genes remain to be delineated across different populations and regions.

**Figure 5:**
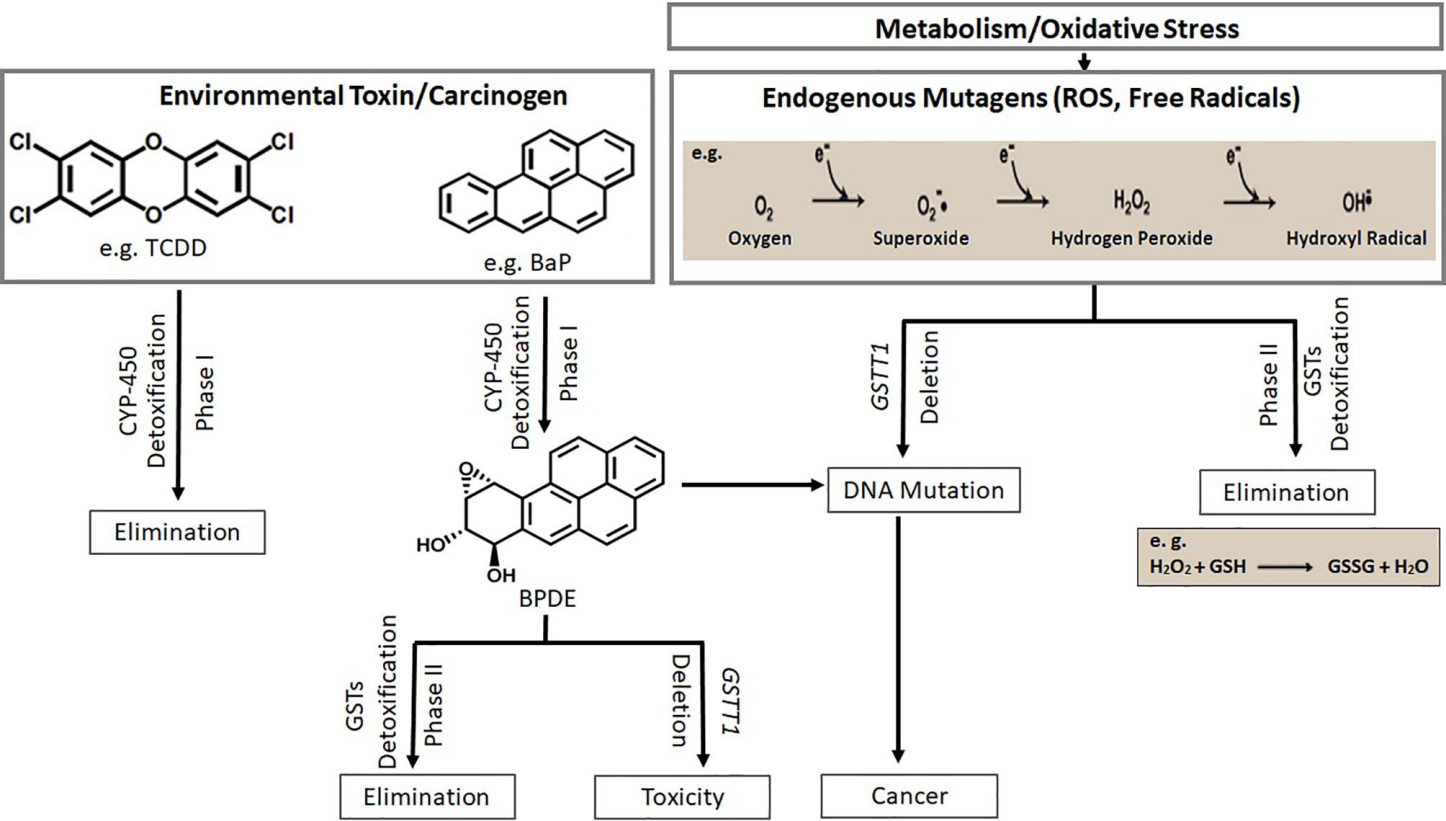
Xeno- and endo-biotic carcinogen metabolism through two pathway systems (phase I and phase II) is shown. Deletion of *GSTT1* leads to toxicity and carcinogenesis (TCDD, 2,3,7,8-Tetrachlorodibenzodioxin; BaP, Benzo[a]pyrene; BPDE, Benzo(a)pyrene diolepoxide; ROS, Reactive Oxygen Species).

A proposed mechanism of carcinogenesis due to the loss of function of GSTT1 isoenzyme is shown in **Figure 5**. Some of the exogenous and endogenous carcinogens are not metabolized to non-toxic components. Consequently, tumorigenesis and/or tumor progression are likely to occur (4). In addition, chemotherapeutic agents may also be metabolized by the pathways involving GSTT1, rendering the patients with *GSTT1*-present genotype irresponsive to either therapy or specific doses of therapy.

Among different populations, the loss-of-function polymorphism in the *GSTT1* encoding gene occurs at varying frequencies (49). Null genotype is correlated with vulnerability to cancers, tumor characteristics, and differences in treatment response (1, 50).

The examples of low and high frequencies of *GSTT1*-absent genotype across specified global regions according to WHO are listed in **Table 5**.

**Table 5:** Distribution of *GSTT1*-absent genotype in different regions across the globe.

Location (Population)	Frequency of *GSTT1* Null Genotype	Reference
**Africa**		
Tunisia (central Tunisian)	0.29	(51)
Gambia (Wollof)	0.5	(52)
**South America**		
Mexico (Western Mexican)	0.03	(53)
Paraguay (Ache)	0.18	(54)
**North America**		
USA (Whites)	0.3	(55)
USA (African-Americans)	0.33	(55)
**Europe**		
Athens, Greece (Greek)	0.1	(56)
Italy (Roman)	0.33	(57)
**Asia**		
Haifa, Israel (Druze), Kabul Afghanistan (Pushtun)	0.07	(58, 59)
Seoul, Korea (Koreans)	0.53	(60)
**Oceania**		
Australia	0.17	(61)

In Pakistan, the frequency of *GSTT1* null genotype in healthy individuals has been reported in the range of 0.06 – 0.24 (16–18, 28–38). This wide range may be attributed to limited sample sizes, population admixture, and differences in methodologies. Similarly, variations in the frequency of this genotype in breast cancer patients from Pakistan have been reported as 8% (16) and 27% (erroneously reported as 49% in the text) (17). The present study reports a frequency of 21%. In contrast to the studies conducted in Punjab/Central Pakistan (16, 17), the present study was carried out in Southern-Pakistan, where the majority of the patients belong to Sindhi and other self-defined Urdu-Speaking ethnicities (25% each). Interestingly, Her2 +ve invasive ductal carcinomas were more frequent in *GSTT1*-absent genotype patients (although data is preliminary, which is available for only 59 samples). Our case-control analysis is in agreement with a number of other studies reported from different parts of the world (11, 62). However, it is the first report of significant association of *GSTT1*-absent genotype with decreased OS in primary breast cancer patients. An earlier study from China reported such an association with untreated metastatic breast cancers (63).

The strength of our study is the underlying unique population, for whom molecular data for breast cancer risk and clinical parameters are scarce. The limitations of the study are sample size and the paucity of information for known risk factors and clinical parameters, primarily due to the restriction of resources in healthcare. In terms of the limitation of the sample size, it should be emphasized that the results reported in the present study do not corroborate previously published reports from this region with the same limitation. The present study also notes that Chi-squared goodness-of-fit test was inappropriate for the association analysis as reported in earlier studies. The methodology results in a lack of data for heterozygous controls. This information is necessary to estimate HWE and subsequent association evaluation.

During the analysis, the present study takes into account missing information for risk factors and clinical parameters as illustrated in **Table 1** and the results section. The *post-hoc* power analysis showed a value of 60.1%. However, it is pertinent to mention here that the calculated estimate does not capture the true power of the study (41). A three-year follow-up provides useful information for assessing the OS in breast cancers in Pakistan. The mortality rate (ASR: 18.8 per 100 000) attributed to breast cancer in the country is among the highest in Asia (15). As emphasized earlier, the reasons behind such a high mortality rate need to be investigated. The significant association of the investigated polymorphism with OS in breast cancers may aid in taking precautionary measures in treatment regimens early in the administration of therapy administration. This study highlights the importance of conducting rigorous molecular epidemiology studies to devise evidence-based better strategies in breast cancer management, particularly in resource-limited settings.

## Conclusions

The present study reports significant contribution of the *GSTT1*-absent genotype to breast cancer risk in a Pakistani population for the first time. A unique finding of this study was the association of this genotype with significantly shorter OS in breast cancer patients post standard treatment, which has not been reported previously. These observations are biologically plausible. If validated further through multiple center studies and larger sample sizes, the absence of the *GSTT1* gene could serve as a risk and survival marker in breast cancers, at least for a specific population.

## Data Availability Statement

The raw data supporting the conclusions of this article is available as the **Supplementary Material**, without any reservations. The sample IDs are confidentially encoded and untraceable.

## Ethics Statement

The studies involving human participants were reviewed and approved by the International Center for Chemical and Biological Sciences (ICCBS), University of Karachi, Karachi, Pakistan [ICCBS/IEC-016-BS/HT-2016/Protocol/1.0]; the Atomic Energy Medical Centre (AEMC), Jinnah Postgraduate Medical Centre (JPMC), Karachi, Pakistan [Admin-3 (257)/2016]; and the Sindh Institute of Urology and Transplantation (SIUT), Karachi, Pakistan. All the participants signed a written informed-consent form prior to sampling. The patients/participants provided their written informed consent to participate in this study.

## Author Contributions

Conceptualization: SA. Participant enrollment/Data collection: SA, S-E-ZZ, SMA, AS, MM, AA, SF, and SK. Benchwork: SA, S-E-ZZ, SMA, AS, AA, and SF. Analysis: SA. Original draft: SA, S-E-ZZ, and SMA. Reviewing and editing: SA. All authors contributed to the article and approved the submitted version.

## Conflict of Interest

The authors declare that the research was conducted in the absence of any commercial or financial relationships that could be construed as a potential conflict of interest.

## Publisher’s Note

All claims expressed in this article are solely those of the authors and do not necessarily represent those of their affiliated organizations, or those of the publisher, the editors and the reviewers. Any product that may be evaluated in this article, or claim that may be made by its manufacturer, is not guaranteed or endorsed by the publisher.
